# Supplementary material for: Wolbachia infections in natural Anopheles populations affect egg laying and negatively correlate with Plasmodium development
Source: Nat Commun. 2016 May 31;7:11772. doi: 10.1038/ncomms11772 (PMC4895022; doi:10.1038/ncomms11772)
Supplement: Supplementary Information — Supplementary Figures 1-3, Supplementary Tables 1-2, Supplementary References [file ncomms11772-s1.pdf]

SUPPLEMENTARY INFORMATION

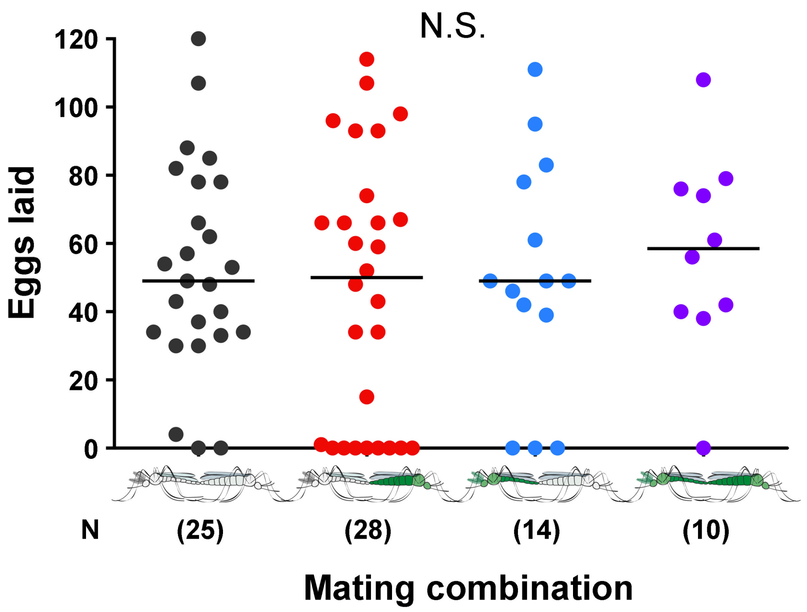

Supplementary Figure 1 | Eggs laid by females in crosses between *w*Anga-infected and uninfected mosquitoes. The number of eggs laid by each female was determined by counting eggs laid on filter papers in each oviposition cup 4 days after egg laying (Kruskal-Wallis, $\chi^2 = 0.686$, d.f. = 3, $p > 0.05$). *w*Anga-infected (green) and uninfected (grey) females (right) and males (left) were identified by 16S nested PCR post-hoc, and mating couples were divided into 4 groups (*w*Anga-negative couples, dark grey; *w*Anga-positive couples, purple; *w*Anga-positive females mated to *w*Anga-negative males, red; *w*Anga-negative females mated to *w*Anga-positive males, blue).

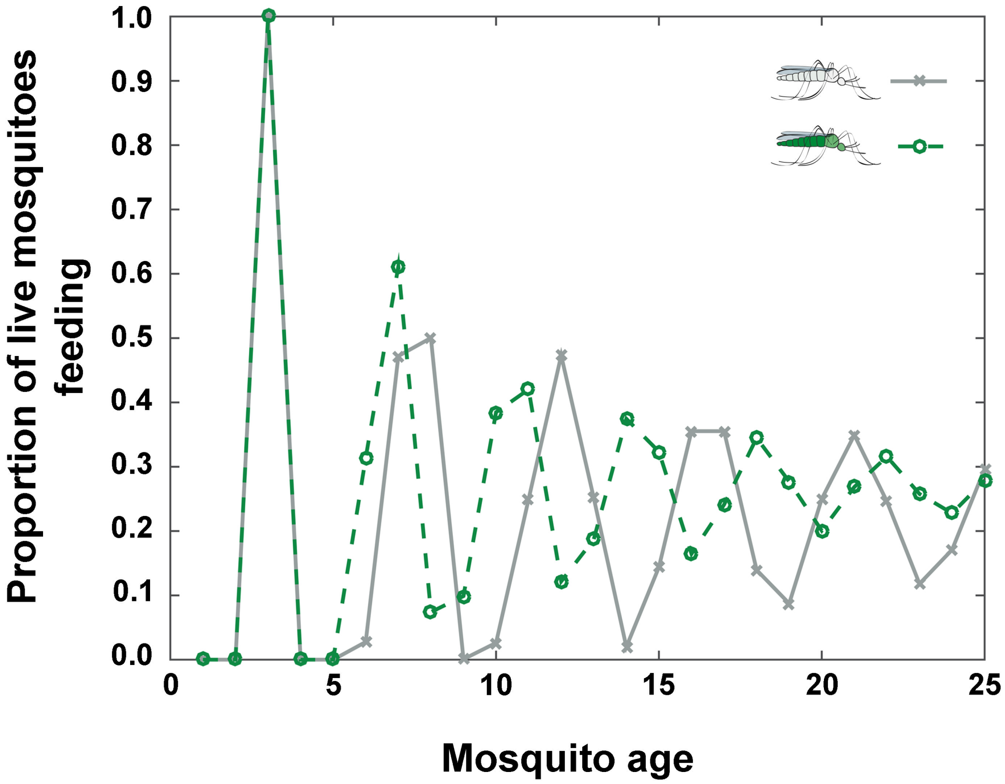

Supplementary Figure 2 | *Wolbachia* infection is predicted to increase the frequency of blood feeding events. The timing of blood-feeds was determined using an individual-based stochastic model with the length of each gonotrophic cycle estimated from the observed time of egg-laying (Figure 2d). Green and grey females and lines indicate *Wolbachia*-infected mosquitoes, respectively. Each curve represents the combination of one million replicate simulations. Assuming a daily adult mortality rate of 0.15 in both groups, the average number of bites over a lifetime is 1.38±1.34 for uninfected females and 1.56±1.57 for *Wolbachia*-infected females. Most mosquitoes bite less than twice in their life span but a few may bite as many as eight times.

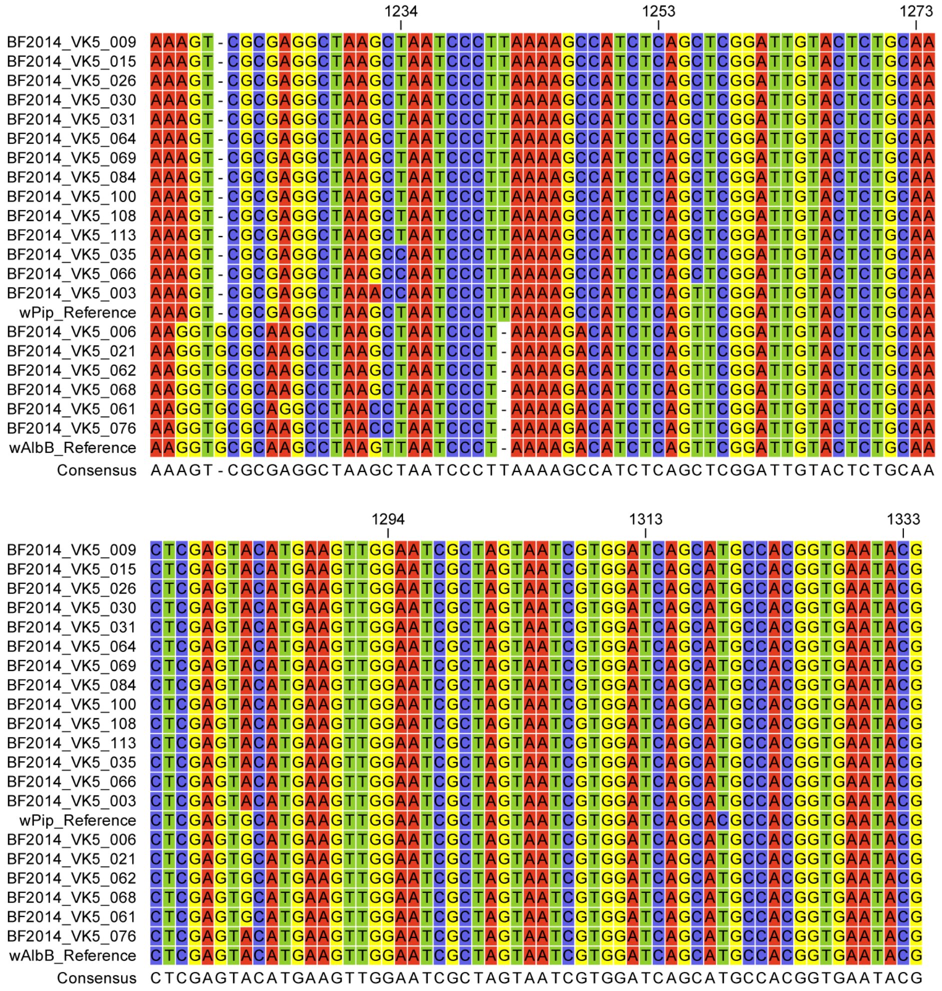

Supplementary Figure 3 | Alignment of *w*Anga 16S rDNA sequences. 221 blood-fed *An. coluzzii* females were collected from the walls of houses in the Vallée du Kou, females were dissected five days later, and the DNA of thoraxes and abdomens was extracted. Using *Wolbachia*-specific primers, the 16S rDNA region of *w*Anga was amplified by nested PCR. The sequence of all PCR-positive samples was determined using Sanger sequencing of the 412 bp product, and all sequencing results were aligned to *w*AlbB (Accession number CAGB01000162) and *w*Pip (Accession number EU096232) reference 16S rDNA sequences (Silva Database). An alignment of a subset of 20 randomly selected sequences from *w*Anga-infected females was constructed using the CLC Sequence Viewer software (CLC Bio, Qiagen). Nucleotides are numbered according to the *w*AlbB 16S rDNA sequence, with the aligned region representing bases 1215-1334.

Year	***An. arabiensis*** (Soumousso)	***An. coluzzii*** (VK5)
**2011**	n.d.	7 / 34 (19%)*
**2013**	16 / 49 (33%)	19 / 91 (21%)*
**2014**	n.d.	275 / 602 (46%)

Supplementary Table 1 | *w*Anga prevalence across several years in Burkina Faso. Mosquito samples collected in two villages (Soumousso and VK5) near Bobo-Dioulasso are shown. Samples from 2011 represent males and females captured in mating swarms, while 2013 samples are blood-fed females collected from the walls of houses. Samples from 2014 represent blood-fed females from houses and also male and female adults raised from field-collected larvae and eggs. * indicates data previously published in Baldini *et al.* 2014 [1].

	***w*Anga +**	***w*Anga –**
***Plasmodium* +**	1 (0.5%)	11 (5.0%)
***Plasmodium* –**	115 (52.0%)	94 (42.5%)

Supplementary Table 2 | *w*Anga and *Plasmodium* prevalence in natural *An. coluzzii* populations in 2014. Samples from 221 blood-fed females collected from houses in VK5 and allowed to develop eggs and oviposit were analyzed for the presence of *w*Anga and *Plasmodium* by 16S nested PCR and 18S qPCR, respectively. The table shows the number of infected females identified for each infection and the percentage of the total. The proportional data is graphed in Figure 3a.

SUPPLEMENTARY REFERENCES

1 Baldini F*, et al.* Evidence of natural *Wolbachia* infections in field populations of *Anopheles gambiae*. *Nature Communications* **5**, 3985 (2014).
